# Self-care behaviors and correlates among adults with type 2 diabetes in Northwest China

**DOI:** 10.3389/fendo.2026.1874726

**Published:** 2026-08-13

**Authors:** Jingjing Pan, Xiaorong Xue, Bin Hu, Lian Wu

**Affiliations:** 1 Department of Pharmacy, Xi’an People’s Hospital (Xi’an Fourth Hospital), Xi’an, China; 2 Department of Ophthalmology, Xi’an People’s Hospital (Xi’an Fourth Hospital), Xi’an, China

**Keywords:** correlates, diabetes mellitus, Northwestern China, self-care, self-management

## Abstract

**Background:**

Poor self-care behaviors remain prevalent among individuals with diabetes worldwide. Although previous Chinese studies examined diabetes self-care, evidence focusing on Northwestern China remains limited. This region’s distinct geographic and lifestyle traits influence patient self-care. This study aimed to assess self-care behaviors among patients with type 2 diabetes mellitus (T2DM) in Northwestern China and identify factors affected with different domains of self-care.

**Methods:**

A hospital-based cross-sectional study with convenience sampling was conducted at Xi’an People’s Hospital (Xi’an Fourth Hospital) from February to August 2025. Eligible inpatients diagnosed with T2DM during the study period were consecutively invited to participate. A total of 410 adult patients were enrolled. The Chinese version of the summary of diabetes self-care activities (SDSCA) scale was used to evaluate patients’ self-care status. Multiple linear regression analysis was employed to identify the factors associated with self-care behaviors.

**Results:**

Overall diabetes self-care among participants was suboptimal (mean 36.11 ± 7.84). Among four self-care domains, physical exercise and foot care showed the poorest adherence, whereas dietary management and glucose monitoring were relatively better maintained. Age (B = 0.096, 95% CI: 0.004-0.189, *P* = 0.041) and education level (B = 2.094, 95% CI: 0.481-3.708, *P* = 0.011) were independently associated with overall self-care behaviors. For specific domains, better dietary self-care was correlated with older age (B = 0.073, 95% CI: 0.027-0.119, *P* = 0.002), higher education level (B = 1.263, 95% CI: 0.457-2.070, *P* = 0.002), and fewer chronic comorbidities (B = -0.651, 95% CI: -1.167 -0.135, *P* = 0.014). Occupational status was associated with exercise self-care; retired patients showed better exercise adherence than employed or unemployed patients (B = 1.067, 95% CI: 0.184-1.951, *P* = 0.018). Higher education was independently associated with better foot care behaviors (B = 0.647, 95% CI: 0.148-0.145, *P* = 0.011).

**Conclusion:**

T2DM Patients in Northwestern China exhibit unsatisfactory overall self-care, especially for foot care and physical exercise. Advanced age and higher education correlated with better self-care outcomes. These results support targeted health education and interventions focused on younger patients and less educated patients.

## Introduction

The prevalence of type 2 diabetes mellitus (T2DM) has reached epidemic proportions globally, representing a major public health challenge. Approximately 11.1% of adults aged 20–79 years, or roughly 1 in 9 individuals, are living with diabetes, and more than 40% of affected individuals remain undiagnosed. By 2059, an estimated 853 million people (about 1 in 8 adults) will have diabetes worldwide (1). In China, the situation is particularly severe. With over 118 million patients, China has the largest diabetes population globally, creating immense challenges for the prevention and management of the disease and its complications (2). Diabetes substantially increases the risk of microvascular and macrovascular complications, including cardiovascular disease, neuropathy, retinopathy and nephropathy (3–5). Such complications not only impair patients’ quality of life but also place a substantial and growing economic burden on families and national healthcare systems (6, 7).

Optimal self-care, including consistent lifestyle modifications and medication adherence, plays a critical role in preventing complications and enhancing quality of life (8). Self-care is defined as patients’ autonomous actions to maintain health and prevent complications. It reflects patient’s daily experiences and choices in managing their condition. It includes adhering to prescribed diets, engaging in regular physical activity, self-monitoring blood glucose (SMBG), performing regular foot examinations, and following a strict medication regimen (1). By practicing recommended self-care behaviors, patients can improve health outcomes, reduce the risk of diabetes complications, and alleviate the burden on healthcare systems (9). In contrast, inadequate diabetic self-care is associated with higher morbidity and mortality, as well as increased medical costs and caregiver workload (10).

Although the importance of self-care is well recognized, inadequate self-care behaviors remain alarmingly prevalent worldwide. Studies have reported that only 32.8% of patients in Southern Brazil adhered to self-care recommendations, with just 23.2% achieving adequate glycemic control (11). Similarly, the vast majority of patients (90.1%) in Saudi Arabia exhibited poor self-care behaviors, while fewer than half (46.7%) of participants in Ethiopia reported good self-care practices (12, 13). A study conducted in Germany revealed that 42.8% of participants maintained consistent self-care behaviors (14). In China, a survey reported that nearly 70% of participants did not perform regular foot examinations, and 80.0% did not monitor their blood glucose daily (15).

A range of factors have been linked to suboptimal adherence to self-care behaviors. Existing studies have identified associations between self-care practices and multiple determinants, including demographic characteristics such as age, sex, and educational attainment, as well as psychological distress and social support levels (16, 17). Furthermore, individual self-care behaviors are also shaped by socioeconomic status, regional characteristics, and cultural norms and customs (18).

Our study was conducted at a tertiary hospital in Xi’an, the capital of Shaanxi province in Northwest China. Cultural values, beliefs, and dietary habits vary widely across China and strongly influence self-care behaviors. In Northwest China, staple foods account for 37.5% of total dietary consumption and are dominated by wheat. Compared with the Chinese dietary guidelines, local residents have insufficient intake of whole grains, beans, animal-sourced foods, eggs, fruits, and vegetables (19). Such dietary patterns may create practical challenges for carbohydrate restriction. Moreover, China exhibits considerable regional economic disparities; cities in the northwest generally have relatively lower levels of economic development compared with those in eastern and southern regions, which may indirectly restrict access to healthcare resources and consequently affect diabetes self-care practices.

Although several studies have explored self-care behaviors among patients with diabetes in China, research focusing on the northwestern regions - where self-care practices have distinct regional characteristics - remains notably scarce. To address this gap, this study aims to examine self-care behaviors and their associated factors among patients with T2DM in Xi’an, Northwestern China.

## Methods

### Study design and participants

This study employed a hospital-based cross-sectional design with convenience sampling. It was conducted at Xi’an People’s Hospital (Xi’an Fourth Hospital) in Xi’an, China, from February and August 2025. Inpatients diagnosed with T2DM who met the inclusion criteria during the study period were consecutively recruited and invited to participate. The inclusion criteria were as follows: (1) aged ≥18 years; (2) clinically diagnosed with T2DM and receiving oral antidiabetic drugs and/or insulin therapy; and (3) voluntary participation in the study. The exclusion criteria were: (1) presence of severe or terminal illness; (2) inability to communicate due to physical or cognitive impairment; and (3) pregnancy.

The minimum sample size was calculated using the formula: s = z^2^[p(1-p)]/d^2^ (20), where s is the minimum required sample size, z is the standard normal variate, p is an estimated proportion of patients with optimal diabetes self-care, and d is the sample error to be tolerated. The confidence interval is 5%; therefore, d is 0.05, z is 1.96. The value of p was set at 38% according to a previous Chinese study (21). As a result, the minimum required sample size was 363.

**Figure 1:**
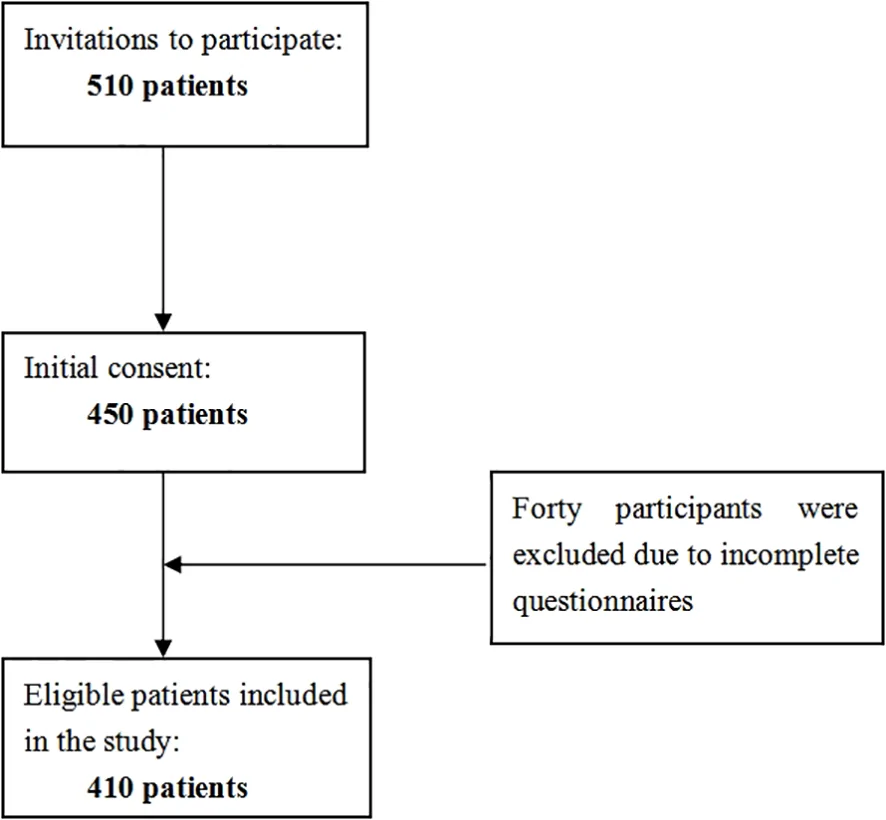
Flowchart of inclusion of participants.

A total of 510 patients were invited to take part in this study, among whom 450 initially consented. Forty participants were subsequently excluded due to incomplete questionnaires caused by on-site interruptions. Finally, 410 eligible patients were included in this study (**Figure 1**).

### Data collection

Prior to questionnaire distribution, investigators explained the study objectives and delivered standardized instructions. Verbal informed consent was obtained from each participant before data collection. Most patients completed the questionnaires independently. For participants unable to read Chinese, trained investigators read each questionnaire item to assist them. All interviewers completed unified standardized training before data collection to ensure consistent survey administration, uniform interpretations of ambiguous items, and standardized communication manners, eliminating leading questions or suggestive prompts that could bias participants’ responses. To protect participant privacy, all one-on-one assisted interviews were carried out in an independent quiet ward room with no additional personnel present. Participants were informed that all personal identifying information would be anonymized during data analysis, and they retained the right to withdraw from the study at any time without adverse consequences.

Sociodemographic data (age, sex, occupation, medical insurance) were obtained from electronic medical records. Trained clinical pharmacists collected additional information via interviews, including residence, education level, living conditions and clinical data (duration of T2DM and the number of other chronic diseases) unavailable in the electronic medical system.

### Instruments

The original summary of diabetes self-care activities (SDSCA) scale evaluates five domains of diabetes management: general diet, specific diet, exercise, medication adherence, and blood-glucose testing. However, the revised SDSCA removed medication-related items due to prominent ceiling effects and narrow response variability, factors that impaired its test-retest reliability in prior research practice (22). The Chinese version of revised SDSCA adopted in the present study contains 10 items across four subscales: diet (4 items), exercise (2 items), blood glucose testing (2 items), foot care (2 items). The Chinese revised SDSCA has undergone formal validation and demonstrates satisfactory reliability and validity (23).

Participants were asked to report the number of days they performed each self-care behavior in the past seven days. All items were scored on a 0-7 point scale, with one point assigned for each day of adherence. Item 4 required reverse scoring prior to total score calculation. Higher scores indicated better self-care adherence. As recommended, weekly adherence days were stratified as follows: fewer than 3 days = poor self-care performance, 3–4 days = moderate self-care performance, and ≥5 days = good self-care performance (24–26). The Cronbach’s alpha coefficient of the scale in our sample was 0.743.

### Data analysis

Descriptive statistics were used to describe the demographic and clinical characteristics of the participants. Categorical variables are presented as frequencies and percentages (%), while continuous variables are summarized as mean ± standard deviation. The chi-square test was performed to compare SDSCA adherence levels between participants with low weekly adherence frequency (<3 days/week) and high weekly adherence frequency (≥5 days/week).

Multiple linear regression analysis was utilized to identify independent correlates of self-care behaviors. Candidate variables entering the regression model were selected according to established clinical relevance and findings from prior peer-reviewed literature to ensure the rationality and interpretability of the model. All categorical predictive variables were dummy coded with clear reference groups prior to model inclusion. Prior to model construction, all core assumptions for linear regression were systematically verified. Multicollinearity among predictors was evaluated using the variance inflation factor (VIF) to guarantee model stability. The adjusted R² was adopted to assess the goodness-of-fit of the final regression model and prevent overestimation driven by redundant variables. Unstandardized beta coefficients (B), 95% confidence intervals (95% CIs), and corresponding P-values were reported for all predictive factors. A significance level of *P* < 0.05 was used to indicate statistical significance. Data analysis were performed using SPSS version 19 (IBM Corp., Armonk, NY, USA).

### Ethical approval

The study was approved by the ethics committee of Xi’an People’s Hospital (Xi’an Fourth Hospital). This research strictly adhered to the ethical principles outlined in the Declaration of Helsinki and complied with relevant Chinese clinical research regulations.

## Results

Of the 410 participants enrolled in this study, 246 (60.00%) were male and 164 (40.00%) were female. A total of 167 participants (40.73%) were aged over 65 years. In terms of employment status, 148 (36.10%) were unemployed, 110 (26.83%) were currently employed, and 152 (37.07%) were retired. A total of 356 participants (86.83%) had medical insurance, whereas only 54 (13.17%) were uninsured. Overall, 304 participants (74.15%) lived in urban areas and 106 (25.85%) lived in rural areas. A total of 251 participants had a high school education or below, accounting for 61.22% of the total sample. Sixteen participants (3.90%) lived alone, 293 (71.46%) lived with their spouses, and 101 (24.63%) lived with their children. In addition, 218 participants (53.17%) had been diagnosed with T2DM for more than 10 years, and 285 participants (69.51%) had one or more comorbid chronic conditions (**Table 1**).

**Table 1:** Sociodemographic and Clinical Characteristics of T2DM Patients (n=410).

Characteristics	Frequency (n)	Percentage(%)
Gender		
Male	246	60
Female	164	40
Age		
<45	40	9.76
45-64	203	49.51
65-79	149	36.34
≥80	18	4.39
Occupation		
Unemployed	148	36.1
Employed	110	26.83
Retired	152	37.07
Health insurance		
Employee medical insurance	47	11.46
Urban resident medical insurance	309	75.37
No medical insurance	54	13.17
Residence		
Urban	304	74.15
Rural	106	25.85
Living condition		
Live alone	16	3.9
Live with spouse	293	71.46
Live with children	101	24.63
Education level		
Primary	72	17.56
High school	179	43.66
College/University	159	38.78
Duration of T2DM (years)		
<5	116	28.29
5-9	76	18.54
10-19	140	34.15
≥20	78	19.02
Number of other chronic diseases		
0	125	30.49
1	171	41.71
≥2	114	27.8

The overall mean score of self-care behaviors was 36.11 ± 7.84. The mean scores across the four subdimensions were 17.84 ± 3.95 for dietary management, 4.95 ± 2.73 for physical exercise, 9.68 ± 3.15 for blood glucose self-monitoring, and 3.63 ± 2.38 for foot care. Overall, physical exercise and foot care were the suboptimal self-care domains. In terms of dietary management, 51.95% of participants adhered to healthy dietary plans for at least 5 days per week; 65.85% ate no fewer than five servings of fruits and vegetables ≥ 5 days weekly, whereas 68.78% consumed high-fat foods (e.g., red meat, full-fat dairy products) on no fewer than 5 days per week. For physical exercise, 46.34% completed at least 30 minutes of physical activity fewer than 3 days per week, and 71.71% engaged in a specific exercise session (e.g., walking, swimming, cycling) fewer than 3 days per week. Regarding blood glucose self-monitoring, 64.39% performed regular blood glucose testing at least 5 days per week, while 59.76% checked their blood glucose in line with medical recommendations for a minimum of 5 days weekly. Foot care was the poorest-performing dimension of the four domains: 72.68% examined their feet fewer than 3 days per week, and 77.32% checked the inside of their shoes fewer than 3 days per week (**Table 2**).

**Table 2:** Frequency of adhering to self-care behavior.

Self-care behaviors (0-7 days)	Average Score (Mean ± SD)	<3 days / week (n,%)	3-4 days/ week (n,%)	≥ 5 days / week (n,%)	*P* value (<3 vs.≥5 days/week)
Total self-care behavior	36.11 ± 7.84				
Diet	17.84 ± 3.95				
Followed a healthful eating plan	4.46 ± 1.38	30 (7.32%)	167 (40.73%)	213 (51.95%)	<0.001
Followed the dietary guidelines per week last month	3.58 ± 1.40	92 (22.44%)	202 (49.27%)	116 (28.29%)	0.096
Ate five or more servings of fruits and vegetables	4.77 ± 1.22	15 (3.66%)	125 (30.49%)	270 (65.85%)	<0.001
Ate high fat foods (red meat or full-fat dairy products)	5.04 ± 1.53	31 (7.56%)	97 (23.66%)	282 (68.78%)	<0.001
Exercise	4.95 ± 2.73				
Participated in at least 30 minutes of physical activity	2.90 ± 1.69	190 (46.34%)	156 (38.05%)	64 (15.61%)	<0.001
Participated in a specific exercise session (such as swimming, walking, biking)	2.06 ± 1.33	294 (71.71%)	89 (21.71%)	27 (6.59%)	<0.001
Self-monitoring of blood glucose	9.68 ± 3.15				
Tested your blood sugar	4.96 ± 1.56	28 (6.83%)	118 (28.78%)	264 (64.39%)	<0.001
Tested your blood sugar the number of times recommended by your health care provider	4.73 ± 1.63	41 (10.00%)	124 (30.24%)	245 (59.76%)	<0.001
Foot care	3.63 ± 2.38				
Checked your feet	1.90 ± 1.26	298 (72.68%)	95 (23.17%)	17 (4.15%)	<0.001
Inspected the inside of your shoes	1.73 ± 1.18	317 (77.32%)	84 (20.49%)	9 (2.20%)	<0.001

Age and educational attainment were independently associated with overall diabetes self-care behaviors. Specifically, total self-care scores rose with advancing age (B = 0.096, 95% CI: 0.004-0.189, *P* = 0.041) and higher educational attainment (B = 2.094, 95% CI: 0.481-3.708, *P* = 0.011). Among the four self-care subscales, dietary self-care was significantly associated with age (B = 0.073, 95% CI: 0.027-0.119, *P* = 0.002), educational attainment (B = 1.263, 95% CI: 0.457-2.070, *P* = 0.002), and the number of comorbid chronic conditions (B = -0.651, 95% CI: -1.167 -0.135, *P* = 0.014). Dietary self-care performance improved with greater age and higher education, yet declined as the count of comorbid chronic illnesses increased. Exercise-related self-care was associated with employment status: retired participants demonstrated better adherence than employed or unemployed individual (B = 1.067, 95% CI: 0.184-1.951, *P* = 0.018). Educational level emerged as an independent positive correlate of foot care behaviors (B = 0.647, 95% CI: 0.148-1.145, *P* = 0.011) (**Table 3**). By contrast, no statistically significant independent predictors were detected for blood glucose self-monitoring.

**Table 3:** Multiple linear regression analysis of factors associated with self-care in T2DM patients.

Variables	Overall Self-care behaviors	Diet	Exercise	Blood glucose monitoring	Foot care
	B (95%Cl)	B (95%Cl)	B (95%Cl)	B (95%Cl)	B (95%Cl)
Gender					
Male	1	1	1	1	1
Female	0.873(-0.765,2.510)	0.646(-0.172,1.464)	0.151(-0.426,0.728)	-0.091(-0.763,0.581)	0.166(-0.340,0.672)
Age	**0.096(0.004,0.189)** ^*^	**0.073(0.027,0.119)** ^**^	0.006(-0.026,0.039)	0.015(-0.023,0.053)	0.003(-0.026,0.031)
Occupation					
Unemployed	1	1	1	1	1
Employed	1.963(-0.747,4.672)	1.021(-0.333,2.375)	0.392(-0.563,1.347)	0.794(-0.318,1.905)	-0.245(-1.082,0.592)
Retired	1.499(-1.008,4.006)	0.005(-1.248,1.257)	1.067(0.184,1.951)*	0.942(-0.086,1.971)	-0.515(-1.290,0.260)
Health insurance					
Employee medical insurance	1	1	1	1	1
Urban resident medical insurance	1.777(-0.772,4.326)	0.797(-0.477,2.071)	0.377(-0.521,1.276)	0.507(-0.538,1.553)	0.095(-0.692,0.883)
No medical insurance	-0.515(-3.757,2.728)	-0.419(-2.040,1.201)	-0.179(-1.322,0.963)	0.407(-0.923,1.737)	-0.323(-1.325,0.679)
Residence					
Urban	1	1	1	1	1
Rural	0.534(-1.746,2.813)	0.192(-0.947,1.331)	0.073(-0.731,0.876)	0.099(-0.836,1.034)	0.170(-0.534,0.874)
Living condition					
Live alone	1	1	1	1	1
Live with spouse	0.251(-3.730,4.233)	0.636(-1.354,2.626)	-1.019(-2.422,0.384)	-0.571(-2.204,1.062)	1.205(-0.025,2.436)
Live with children	0.766(-3.493,5.026)	0.913(-1.216,3.042)	-1.110(-2.611,0.391)	-0.137(-1.885,1.610)	1.101(-0.215,2.417)
Education level	**2.094(0.481,3.708)** ^*^	**1.263(0.457,2.070)** ^**^	0.117(-0.452,0.686)	0.067(-0.595,0.729)	**0.647(0.148,1.145)** ^*^
Duration of T2DM (years)	-0.029(-0.130,0.071)	0.024(-0.026,0.075)	-0.021(-0.057,0.014)	-0.031(-0.072,0.010)	-0.002(-0.033,0.029)
Number of other chronic diseases	-0.908(-1.941,0.124)	**-0.651(-1.167,-0.135)** ^*^	-0.096(-0.460,0.268)	-0.024(-0.447,0.400)	-0.137(-0.457,0.182)

Bold values indicate statistically significant differences (P < 0.05). * P < 0.05, ** P < 0.01.

## Discussion

Diabetes is widely recognized as a chronic condition requiring long-term self- management, with patients bearing primary responsibility for their own health maintenance. Consistent adherence to self-care behaviors constitutes a core pillar of comprehensive diabetes management (27). This study explored the current status of diabetes self-care practices among patients in Northwest China and identified significant correlations between multiple sociodemographic factors and adherence to diabetes self-care regimens.

### Overall self-care behaviors

In the present study, the overall mean SDSCA score was 36.11 ± 7.84. Given the maximum possible total score of 70, this average value was relatively low, suggesting suboptimal self-care levels among enrolled participants. This finding is consistent with results from several previous studies (28, 29), which further illustrates that inadequate diabetes self-care remains a major public health burden across China and worldwide.

Notable disparities existed across the four self-care subscales. Foot care and physical exercise produced the lowest mean scores, while dietary management and blood glucose self-monitoring achieved comparatively higher scores in our sample. The present results partially align with prior research yet reveal several divergent trends.

One earlier study reported similar findings, indicating that a substantial proportion of patients did not engage in regular exercise and exhibited poor foot care adherence (30). In contrast, another domestic Chinese study reported the lowest mean scores for foot care and blood glucose monitoring, while exercise and dietary behaviors obtained relatively higher scores (31). One additional study documented the highest score for foot care, followed by blood glucose monitoring, exercise, and diet (26). Such discrepancies may stem from differences in study populations, cultural backgrounds, and socioeconomic conditions across regions.

Age and educational level were independently associated with the overall self-care practices among patients with T2DM, with better self-care adherence correlated with higher educational attainment and advanced age in this study. By contrast, a previous Chinese study of 163 diabetic patients identified low education and advanced age as predictors of inadequate self-care (32). In addition, distinct independent correlates were detected for each of the four self-care subscales: diet, exercise, foot care, and blood glucose monitoring.

### Diet

Regarding dietary self-care, the mean score was relatively higher among the four dimensions of self-care behavior. The finding is consistent with previous studies, in which diet was generally one of the better-performing aspects of self-care (33). In addition, 65.85% of participants consumed five or more servings of fruits and vegetables for at least five days per week, while 68.78% ate high-fat foods such as red meat and full-fat dairy products for five or more days per week in this study. Although most participants maintained adequate fruit and vegetable intake, their consumption of high-fat foods was excessive. These findings highlight the need for further patient education on diabetes-specific dietary management.

Age, educational level, and the number of comorbid chronic diseases were identified as independent correlates of dietary self-care. Dietary self-care behaviors appeared to be better among participants with older age and higher educational attainment. This finding is consistent with several previous studies showing that older patients demonstrate greater adherence to dietary recommendations for diabetes (30, 34).

One possible explanation is that the longer individuals live with the disease, the more conscious and aware they become of their health, which could lead them to pay greater attention to healthy dietary habits. In addition, many older patients are retired and free from work-related stress, which may afford them more time to plan and arrange their meals. In contrast, younger patients, who generally have a shorter duration of diabetes and fewer comorbid conditions, tend to pay less attention to their self-care practices. Therefore, it is of great importance for young patients with diabetes to recognize the need for self-care. As young adults may develop diabetes-related complications as early as their 20s, identifying strategies and interventions to improve self-care in this population is crucial (35).

Educational level was also positively associated with dietary self-care behaviors in our study. This is consistent with a study reporting that years of schooling are positively correlated with consistent adherence to dietary regimens (36). The positive association between educational level and dietary self-care may be partly explained by the fact that individuals with higher educational attainment are more capable of comprehending all components of recommended self-management and possess greater awareness of how healthy nutrition contributes to glycemic control in diabetes management.

Contrary to prior studies showing patients with comorbidities achieved better diabetes self-care than those without chronic coexisting conditions (37, 38), the present study found that dietary self-care declined as the number of comorbidities increased. A plausible explanation is that patients with multiple chronic conditions must manage not only type 2 diabetes but also other disorders, each of which may have different dietary requirements. Consequently, these patients may lack sufficient energy and capacity to simultaneously balance the dietary needs of multiple conditions.

### Exercise

Physical activity serves as a behavioral intervention for patients to manage diabetes. In the present study, nearly half of participants performed at least 30 minutes of physical activity on fewer than three days per week. Furthermore, 71% of participants took part in specific forms of exercise (e.g., swimming, walking, biking) on fewer than three days weekly. These findings align with prior research noting insufficient regular exercise among diabetes patients and low overall physical activity self-care scores (39). By contrast, another study observed superior exercise self-care, with 55.71% of participants following a fixed exercise schedule for three or more days during the prior week (40).

In this context, retired individuals demonstrated more satisfactory self-care behaviors compared to those who were employed or unemployed. One possible explanation is that retired people might have more time and energy to engage in regular physical activities, whereas working individuals may lack time or experience fatigue after work, which could make consistent exercise challenging. Additionally, it is also plausible that limited exercise-related knowledge and low motivation to exercise may contribute to poorer exercise adherence (41).

### Foot care

Diabetic foot ulcers are a prevalent complication among people living with diabetes and constitute a top cause of diabetes-attributable disability. Proper foot care is essential to the prevent diabetic foot disorders. Nevertheless, prior investigations consistently report suboptimal foot care practices. A Chinese study involving 322 individuals with diabetes revealed that foot care was the least frequently implemented self-care behavior, with 78.9% of participants failing to perform routine foot examinations (42). Likewise, Okafor et al. reported inadequate foot self-care, where only a small minority of patients performing daily foot inspections (24). In contrast, Abdullahi et al. observed comparatively satisfactory foot care adherence, as 82.44% of participants reported regular foot inspections; such high adherence was partly attributed to regional cultural and religious characteristics (43).

In our study, foot care yielded the lowest score across all four self-management domains. Only 4.15% and 2.20% of participants performed foot self-examination and checked the inside of their shoes for five or more days per week, respectively. One reasonable interpretation is that patients may prioritize other aspects of diabetes care over foot screening, viewing the latter as lower priority (44). This finding indicates that patients with T2DM in Northwest China overall have insufficient knowledge about foot protection and diabetes chronic complications.

Additionally, this study observed an association between higher educational attainment and better foot care behaviors. Similar results have been reported by Kassie et al., who found that patients with diabetes who had attended college or above were 4.4 times more likely to practice adequate foot self-care than those with no formal education (38). One plausible explanation is that patients with higher education levels may be more likely to acquire systematic knowledge of T2DM and its complications, including professional foot care guidelines. A higher educational background may correlate with more positive cognition, appropriate health attitudes and standardized disease management practices for diabetes prevention and control, which could facilitate greater familiarity with diabetes guidelines and better adherence to professional medical advice. Currently, targeted health education for diabetic patients remains inadequate. It is reported that those who had poor diabetes knowledge were 5 times more likely to have poor self-care practices than those who had good diabetes knowledge (25). Accordingly, diversified health education strategies should be developed and promoted in clinical practice in the future, with targeted interventions focusing on patients with low educational levels.

### Self-monitoring of blood glucose

Self-monitoring of blood glucose achieved the highest mean score across the four self-management dimensions in this study, with most participants performing blood glucose testing at least five days weekly. This result aligns with prior research demonstrating that 60.8% of adults with diabetes complied with official guidelines for blood glucose self-monitoring (45).

Nevertheless, several prior studies have reported contradictory outcomes. One investigation found that almost all (99.7%) participants had inadequate blood sugar monitoring self-care (46). A Chinese investigation similarly observed lower adherence to blood glucose monitoring compared with other self-care practices (47). Moreover, an additional Chinese study ranked blood glucose testing as the second poorest-performing item on the SDSCA scale, with scores below 2.36 (48).

The relatively high adherence to blood glucose self-monitoring in our research may be partly attributable to elevated health awareness and improved diabetes-related health literacy among local patients. It is possible that more individuals currently follow medical guidance to conduct regular blood glucose testing. Given that blood glucose monitoring has long been a core component of routine diabetes health education, patients are likely to recognize its clinical significance. Furthermore, alongside socioeconomic development and improved living standards, household glucometer ownership may have become more widespread, which could render daily blood glucose monitoring more accessible and convenient.

None of the factors showed significant associations with blood glucose self-monitoring behaviors in the present study, which may be due to differences in research populations, social culture, and the classification and evaluation criteria of self-management behaviors.

Several limitations of this study should be acknowledged. First, this is a single-center, hospital-based cross-sectional design, which limits the external generalizability of our results; accordingly, the findings cannot be fully extrapolated to all patients with T2DM across Northwest China. Second, participants were recruited through convenience sampling, potentially reducing sample representativeness and incurring selection bias. Third, all self-care outcomes were collected via self-reported questionnaires. Self-assessment is susceptible to inherent measurement biases, which may lead to overestimated or underestimated self-care adherence and undermine data objectivity. Lastly, some participants who were unable to read Chinese completed questionnaires with interviewer assistance. While this approach guaranteed effective survey completion, subtle investigator guidance may interfere with participants’ responses, resulting in potential information bias.

## Conclusion

Overall, self-care behaviors remain suboptimal among adults with T2DM in Northwest China. Foot care and physical exercise exhibited the poorest performance across the four evaluated domains. Age and education level were independent correlates of overall self-care performance. These results suggest a need for enhanced diabetes self-care education and support, with particular attention to younger patients and those with lower educational attainment.

## Data Availability

The original contributions presented in the study are included in the article/**Supplementary Material**. Further inquiries can be directed to the corresponding author.
