# Supplementary material for: Self-care behaviors and correlates among adults with type 2 diabetes in Northwest China
Source: Front Endocrinol (Lausanne). 2026 Aug 13;17:1874726. doi: 10.3389/fendo.2026.1874726 (PMC13518134; doi:10.3389/fendo.2026.1874726)

NPar Tests

[DataSet0]

Chi-Square

Frequencies

group=1 or group=3 (FILTER)

	Observed N	Expected N	Residual
Selected	243	243.0	.0
Total	243[a]		

a. This variable is constant. Chi-Square Test cannot be performed.

group

	Observed N	Expected N	Residual
<3	30	121.5	-91.5
>5	213	121.5	91.5
Total	243		

Test Statistics

	group
Chi-Square	1.378E2[a]
df	1
Asymp. Sig.	.000

a. 0 cells (.0%) have expected frequencies less than 5. The minimum expected cell frequency is 121.5.

Chi-Square

Frequencies

group=1 or group=3 (FILTER)

	Observed N	Expected N	Residual
Selected	208	208.0	.0
Total	208[a]		

a. This variable is constant. Chi-Square Test cannot be performed.

group

	Observed N	Expected N	Residual
<3	92	104.0	-12.0
>5	116	104.0	12.0
Total	208		

Test Statistics

	group
Chi-Square	2.769[a]
df	1
Asymp. Sig.	.096

a. 0 cells (.0%) have expected frequencies less than 5. The minimum expected cell frequency is 104.0.

```
NPAR TEST
   /CHISQUARE=filter_$ group
   /EXPECTED=EQUAL
   /MISSING ANALYSIS.
```

Chi-Square

Frequencies

group=1 or group=3 (FILTER)

	Observed N	Expected N	Residual
Selected	285	285.0	.0
Total	285[a]		

a. This variable is constant. Chi-Square Test cannot be performed.

group

	Observed N	Expected N	Residual
<3	15	142.5	-127.5
>5	270	142.5	127.5
Total	285		

Test Statistics

	group
Chi-Square	2.282E2[a]
df	1
Asymp. Sig.	.000

a. 0 cells (.0%) have expected frequencies less than 5. The minimum expected cell frequency is 142.5.

Chi-Square

Frequencies

group=1 or group=3 (FILTER)

	Observed N	Expected N	Residual
Selected	313	313.0	.0
Total	313[a]		

a. This variable is constant. Chi-Square Test cannot be performed.

group

	Observed N	Expected N	Residual
<3	31	156.5	-125.5
>5	282	156.5	125.5
Total	313		

Test Statistics

	group
Chi-Square	2.013E2[a]
df	1
Asymp. Sig.	.000

a. 0 cells (.0%) have expected frequencies less than 5. The minimum expected cell frequency is 156.5.

Chi-Square

Frequencies

group=1 or group=3 (FILTER)

	Observed N	Expected N	Residual
Selected	254	254.0	.0
Total	254[a]		

a. This variable is constant. Chi-Square Test cannot be performed.

group

	Observed N	Expected N	Residual
<3	190	127.0	63.0
>5	64	127.0	-63.0
Total	254		

Test Statistics

	group
Chi-Square	62.504[a]
df	1
Asymp. Sig.	.000

a. 0 cells (.0%) have expected frequencies less than 5. The minimum expected cell frequency is 127.0.

Chi-Square

Frequencies

group=1 or group=3 (FILTER)

	Observed N	Expected N	Residual
Selected	321	321.0	.0
Total	321[a]		

a. This variable is constant. Chi-Square Test cannot be performed.

group

	Observed N	Expected N	Residual
<3	294	160.5	133.5
>5	27	160.5	-133.5
Total	321		

Test Statistics

	group
Chi-Square	2.221E2[a]
df	1
Asymp. Sig.	.000

a. 0 cells (.0%) have expected frequencies less than 5. The minimum expected cell frequency is 160.5.

Chi-Square

Frequencies

group=1 or group=3 (FILTER)

	Observed N	Expected N	Residual
Selected	292	292.0	.0
Total	292[a]		

a. This variable is constant. Chi-Square Test cannot be performed.

group

	Observed N	Expected N	Residual
<3	28	146.0	-118.0
>5	264	146.0	118.0
Total	292		

Test Statistics

	group
Chi-Square	1.907E2[a]
df	1
Asymp. Sig.	.000

a. 0 cells (.0%) have expected frequencies less than 5. The minimum expected cell frequency is 146.0.

Chi-Square

Frequencies

group=1 or group=3 (FILTER)

	Observed N	Expected N	Residual
Selected	286	286.0	.0
Total	286[a]		

a. This variable is constant. Chi-Square Test cannot be performed.

group

	Observed N	Expected N	Residual
<3	41	143.0	-102.0
>5	245	143.0	102.0
Total	286		

Test Statistics

	group
Chi-Square	1.455E2[a]
df	1
Asymp. Sig.	.000

a. 0 cells (.0%) have expected frequencies less than 5. The minimum expected cell frequency is 143.0.

Chi-Square

Frequencies

group=1 or group=3 (FILTER)

	Observed N	Expected N	Residual
Selected	315	315.0	.0
Total	315[a]		

a. This variable is constant. Chi-Square Test cannot be performed.

group

	Observed N	Expected N	Residual
<3	298	157.5	140.5
>5	17	157.5	-140.5
Total	315		

Test Statistics

	group
Chi-Square	2.507E2[a]
df	1
Asymp. Sig.	.000

a. 0 cells (.0%) have expected frequencies less than 5. The minimum expected cell frequency is 157.5.

Chi-Square

Frequencies

group=1 or group=3 (FILTER)

	Observed N	Expected N	Residual
Selected	326	326.0	.0
Total	326[a]		

a. This variable is constant. Chi-Square Test cannot be performed.

group

	Observed N	Expected N	Residual
<3	317	163.0	154.0
>5	9	163.0	-154.0
Total	326		

Test Statistics

	group
Chi-Square	2.910E2[a]
df	1
Asymp. Sig.	.000

a. 0 cells (.0%) have expected frequencies less than 5. The minimum expected cell frequency is 163.0.

Regression

[数据集1] E:\研究\2025SCI2\data2.sav

Variables Entered/Removed

Model	Variables Entered	Variables Removed	Method
1	livewithchildren, urbanresidentinsurance, otherchronicdiseases, residence, durationofT2DM, gender, employed, educationlevel, age, noinsurance, retired, livewithspouse[a]	.	Enter

a. All requested variables entered.

Model Summary[b]

Model	R	R Square	Adjusted R Square	Std. Error of the Estimate	Change Statistics					Durbin-Watson
					R Square Change	F Change	df1	df2	Sig. F Change	
1	.265[a]	.070	.042	7.67035	.070	2.501	12	397	.004	1.815

a. Predictors: (Constant), livewithchildren, urbanresidentinsurance, otherchronicdiseases, residence, durationofT2DM, gender, employed, educationlevel, age, noinsurance, retired, livewithspouse

b. Dependent Variable: VAR00010

ANOVA[b]

Model		Sum of Squares	df	Mean Square	F	Sig.
1	Regression	1765.652	12	147.138	2.501	.004[a]
	Residual	23357.187	397	58.834		
	Total	25122.839	409			

a. Predictors: (Constant), livewithchildren, urbanresidentinsurance, otherchronicdiseases, residence, durationofT2DM, gender, employed, educationlevel, age, noinsurance, retired, livewithspouse

b. Dependent Variable: VAR00010

Coefficients[a]

Model		Unstandardized Coefficients		Standardized Coefficients	t	Sig.	95.0% Confidence Interval for B		Correlations			Collinearity Statistics	
		B	Std. Error	Beta			Lower Bound	Upper Bound	Zero-order	Partial	Part	Tolerance	VIF
1	(Constant)	25.638	3.779		6.785	.000	18.209	33.067					
	gender	.873	.833	.055	1.048	.295	-.765	2.510	.033	.053	.051	.862	1.160
	age	.096	.047	.144	2.054	.041	.004	.189	.043	.103	.099	.474	2.109
	residence	.534	1.159	.030	.460	.646	-1.746	2.813	-.132	.023	.022	.557	1.796
	educationlevel	2.094	.821	.193	2.552	.011	.481	3.708	.168	.127	.123	.411	2.434
	durationofT2DM	-.029	.051	-.030	-.578	.564	-.130	.071	-.011	-.029	-.028	.890	1.124
	otherchronicdiseases	-.908	.525	-.089	-1.729	.085	-1.941	.124	-.044	-.086	-.084	.894	1.119
	employed	1.963	1.378	.111	1.424	.155	-.747	4.672	.031	.071	.069	.385	2.598
	retired	1.499	1.275	.093	1.176	.240	-1.008	4.006	.135	.059	.057	.378	2.644
	urbanresidentinsurance	1.777	1.297	.098	1.370	.171	-.772	4.326	.099	.069	.066	.460	2.176
	noinsurance	-.515	1.649	-.022	-.312	.755	-3.757	2.728	-.121	-.016	-.015	.461	2.168
	livewithspouse	.251	2.025	.015	.124	.901	-3.730	4.233	-.039	.006	.006	.172	5.829
	livewithchildren	.766	2.167	.042	.354	.724	-3.493	5.026	.046	.018	.017	.165	6.074

a. Dependent Variable: VAR00010

Collinearity Diagnostics[a]

Model	Dimension	Eigenvalue	Condition Index	Variance Proportions												
				(Constant)	gender	age	residence	educationlevel	durationofT2DM	otherchronicdiseases	employed	retired	urbanresidentinsurance	noinsurance	livewithspouse	livewithchildren
1	1	7.286	1.000	.00	.00	.00	.00	.00	.00	.00	.00	.00	.00	.00	.00	.00
	2	1.311	2.357	.00	.01	.00	.05	.00	.00	.00	.03	.03	.00	.10	.00	.01
	3	1.150	2.517	.00	.04	.00	.10	.01	.00	.00	.07	.00	.00	.02	.00	.01
	4	.941	2.782	.00	.01	.00	.03	.00	.00	.00	.04	.00	.01	.06	.01	.06
	5	.839	2.948	.00	.08	.00	.02	.00	.01	.00	.05	.08	.01	.12	.00	.01
	6	.465	3.957	.00	.73	.00	.09	.00	.04	.02	.00	.00	.01	.06	.00	.01
	7	.341	4.621	.00	.00	.00	.00	.00	.41	.56	.00	.00	.00	.00	.00	.00
	8	.289	5.021	.00	.03	.00	.20	.03	.38	.31	.00	.10	.00	.00	.00	.00
	9	.174	6.462	.00	.07	.01	.41	.04	.13	.03	.11	.14	.15	.17	.01	.01
	10	.096	8.730	.00	.00	.00	.00	.57	.00	.01	.61	.58	.03	.02	.00	.01
	11	.077	9.746	.01	.02	.02	.01	.09	.01	.02	.00	.00	.61	.37	.14	.11
	12	.023	17.637	.04	.00	.26	.00	.01	.00	.01	.00	.05	.14	.06	.76	.72
	13	.007	32.161	.95	.01	.70	.07	.24	.01	.02	.09	.02	.04	.02	.07	.05

a. Dependent Variable: VAR00010

Residuals Statistics[a]

	Minimum	Maximum	Mean	Std. Deviation	N
Predicted Value	29.5831	41.0065	36.1122	2.07774	410
Residual	-18.44875	22.68948	.00000	7.55699	410
Std. Predicted Value	-3.142	2.356	.000	1.000	410
Std. Residual	-2.405	2.958	.000	.985	410

a. Dependent Variable: VAR00010

Charts

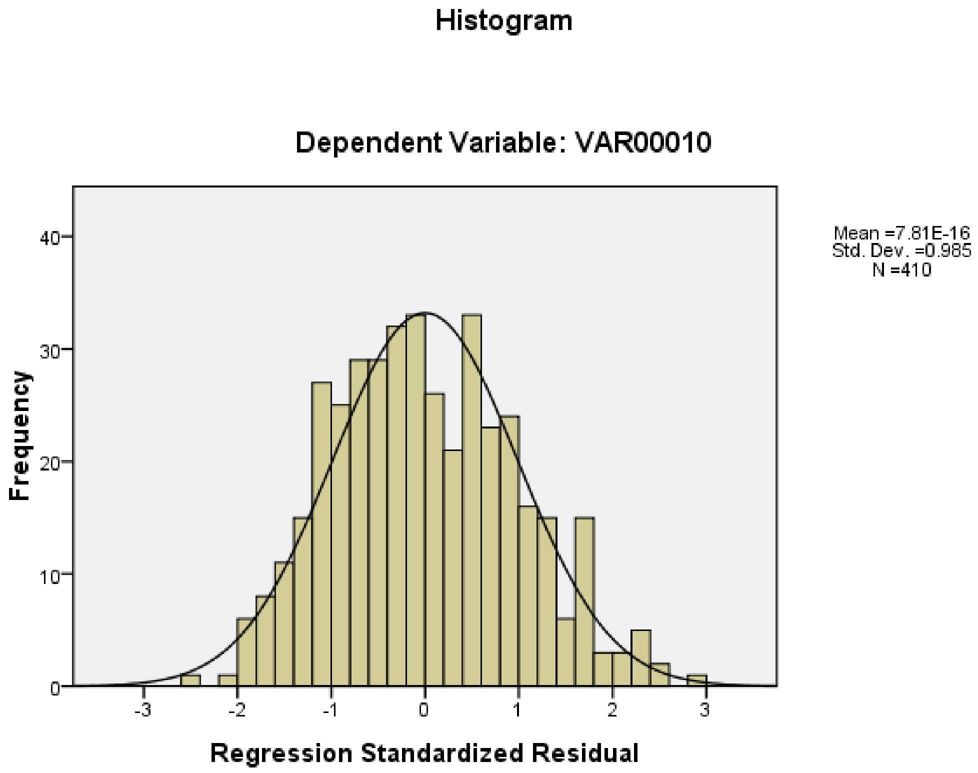

Normal P-P Plot of Regression Standardized Residual

Dependent Variable: VAR00010

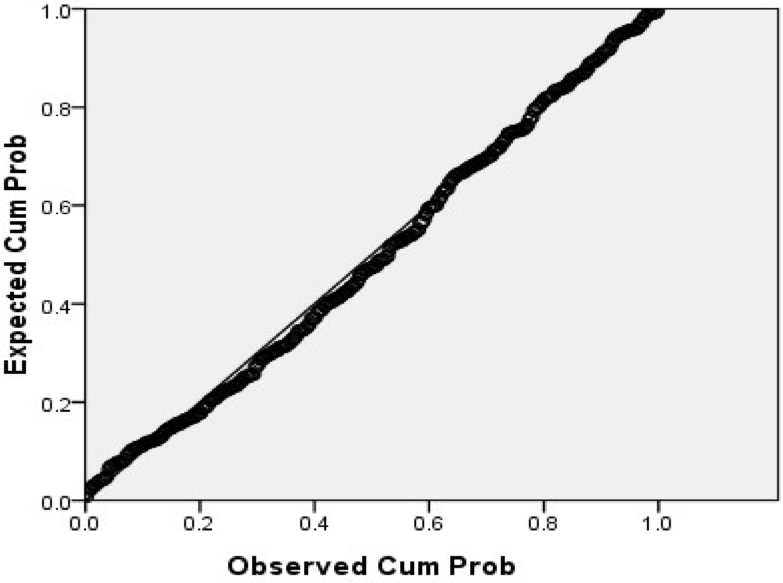

Scatterplot

Dependent Variable: VAR00010

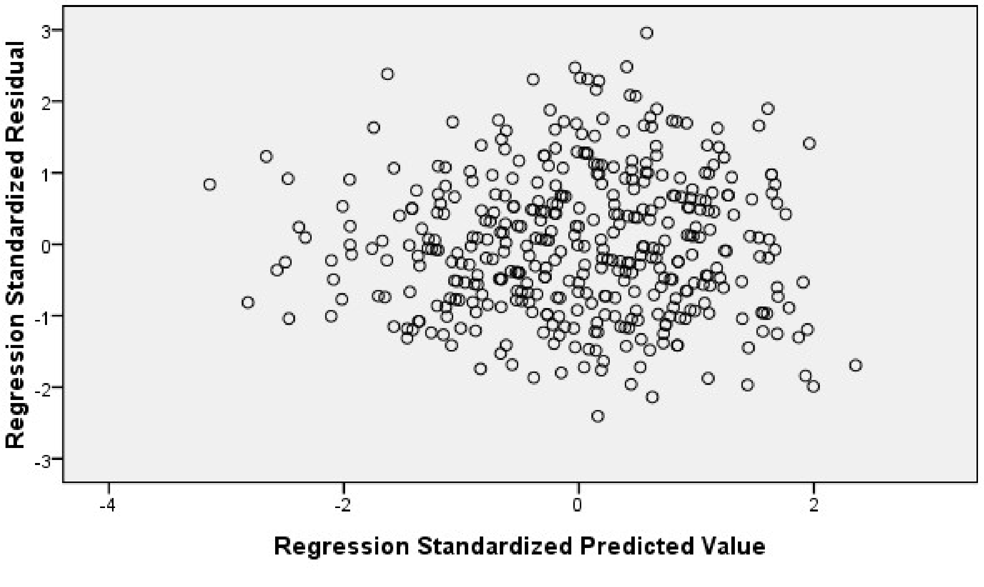

Supplement: Supplementary file 1 [file Table2.docx]
